# Use of multikinase inhibitors/lenvatinib concomitant with radioiodine for the treatment of radioiodine refractory differentiated thyroid cancer

**DOI:** 10.1002/cam4.5105

**Published:** 2022-10-06

**Authors:** Urbano Anido Herranz

**Affiliations:** ^1^ Medical Oncology Department University Hospital of Santiago de Compostela Santiago de Compostela A Coruña Spain

**Keywords:** lenvatinib, multikinase inhibitors, radioiodine‐refractory thyroid cancer, sorafenib

## Abstract

Thyroid cancer is the most frequent endocrine tumor. However, in locally advanced or metastatic disease we have only two types of treatment at our disposal: radioactive iodine (RAI) when the disease is RAI‐sensitive and multikinase inhibitors (MKIs), lenvatinib and sorafenib, when the disease becomes RAI‐refractory (RR). This review revisits the published data on the potential combination of MKIs/lenvatinib with RAI in RR‐differentiated thyroid cancer and evaluates some special situations where this combination may be of particular interest. The combination of MKIs/lenvatinib with RAI could, at least hypothetically, improve the efficacy seen in both treatments alone via a synergistic effect and with a lower rate of toxicity rates. Early preclinical data support this notion, while its generalized use awaits the results of ongoing clinical trials.

## Abstract 2

There are currently only two treatments available for thyroid cancer: radioactive iodine (RAI) for RAI‐sensitive disease and multikinase inhibitors (MKIs) for RAI‐refractory disease. The combination of MKIs/lenvatinib with RAI may improve the efficacy of both treatments via a synergistic effect and with a lower rate of toxicity. The article reviews the published data on this therapeutic combination.

## 1 INTRODUCTION

Thyroid cancer was the 9th most common cancer in 2020 with 586,202 new cases globally^1^ and a small increase in overall mortality.^2^ Locally advanced or metastatic differentiated thyroid cancer (DTC) has only two treatment options, radioactive iodine (RAI) therapy, when the disease is RAI‐sensitive, and multikinase inhibitors (MKIs), when it becomes RAI‐refractory (RR) or does not respond to RAI. Median overall survival of RR‐DTC patients ranged between 39.4 months (95% confidence interval [CI]: 32.7–51.4) seen in the patients treated with sorafenib and 41.6 months (95% CI: 31.2–not estimable) in patients treated with lenvatinib.^3^

Lenvatinib and sorafenib have demonstrated improvement in progression‐free survival (PFS) and overall response rate (ORR) in the SELECT^4^ and DECISION^5^, ^6^ trials, respectively in patients with RR‐DTC. Median PFS and ORR for lenvatinib were 18.3 months and 64.8% while for sorafenib these were 10.8 months and 12.2%. Both trials also reported a significant number of toxicities, however, only 14.2% and 18.8% of patients had to discontinue treatment with lenvatinib or sorafenib due to safety reasons.

The use of MKIs concomitant with RAI for the treatment of RR‐DTC has not yet been explored. Still, there are reasons to believe this combination could improve both the efficacy and toxicity of each agent alone. This paper aims to review the rational and potential role of this strategy for the treatment of RR‐DTC in special circumstances.

## 2 HISTORICAL PERSPECTIVE

The first option for the treatment of advanced DTC is surgery and RAI,^7^ but the natural progression of this disease is to become RAI‐refractory in most cases. At that stage, an available treatment choice is MKIs. Approvals of lenvatinib and sorafenib by FDA and EMA have been an important advance for patients with RR‐DTC despite these trials not demonstrating a comparative increase in overall survival (OS) against their placebo arms. Provided both trials had a high crossover rate (≥75%), it was not possible to directly evaluate the impact these interventions had on the survival expectancy of patients originally randomized to placebo or active treatment. Thus using statistical adjustments to estimate the OS achieved by patients in the placebo arms had they not crossed over to sorafenib,^5^ or lenvatinib,^8^ showed only lenvatinib produced an OS benefit.

Recently, a new phase 3 trial (COSMIC‐311) has been published.^9^ This trial evaluated cabozantinib versus placebo in locally advanced or metastatic RR‐DTC that had progressed to a prior VEGFR‐therapy. Cabozantinib showed a median PFS of 6.2 months for the intention to treat population, granting its approval by the FDA in this pretreated population. Other MKIs are being studied in this setting, however, RR‐DTC remains an orphan disease without good treatment options and still a poor prognosis.

Combination therapies are being explored in other neoplasms as a strategy to improve RAI‐refractory tumors to optimize the available treatment options, while new treatments are being developed.

## 3 CURRENT SITUATION

Today's definition of RR‐DTC has not been free of controversy. The American Thyroid Association^7^ consensus defines RR‐DTC on the basis four premises: (i) the neoplastic tissue does not ever uptake or concentrate RAI; (ii) the tumor tissue loses the capability to concentrate RAI after previous evidence of RAI‐avid disease; (iii) RAI is concentrated in some lesions but not in others, particularly when 2‐deoxy‐2‐[^18^F]fluoro‐D‐glucose positron emission tomography (^18^FDG‐PET) uptake is present; and (iv) metastatic disease progresses despite a significant concentration of RAI and there is a continual rise of tiroglobulin within months of RAI therapy.

Once a DTC patient has been diagnosed RAI‐refractory, current treatment guidelines recommend avoiding further RAI treatment. However, a couple of ongoing initiatives are investigating the combination of lenvatinib with RAI in DTC, one of which is a phase 2 study of this combo in patients with progressive RAI‐sensitive DTC^10^ (NCT03506048). Another one is the RESET trial, that aims at reinducing RAI‐sensitivity in patients with RR‐DTC tumors using Lenvatinib^11^ (NCT04858867). None has yet published results.

The preclinical rationale supporting this research is based on increased apoptosis and cells arresting in the G2/M phase of the cell cycle observed in xenograft murine models, suggesting a synergistic effect of the combination. The increase in intracellular uptake of lenvatinib through augmented cell membrane permeability may explain these apparent synergistic antitumoral effects.^12^ An additional supporting mechanism relies on the vasculature‐normalizing effect commonly seen in antiangiogenic drugs like lenvatinib, that is thought to improve the usual hypoxic tumoral microenvironment, consequently, requiring lower MKI dose and toxicity.^12^ A preclinical trial with anaplastic thyroid cancer cells has demonstrated that K905‐0266 MKI increases 125‐iodine uptake in vitro and in vivo and enhanced cytotoxicity of 131‐iodine therapy.^13^

These observations are in line with the accepted knowledge of RAI efficacy relying on its accumulation in thyroid cancer cells. This has served the basis for the hypothesis sustaining resensitization to RAI may be a promising therapeutic strategy against thyroid cancer. In fact, some data on resensitization to RAI with MKIs in RR‐DTC have been published. One of the first drug groups investigated was retinoids, with retinoic acid producing some and resensitization to RAI,^14^, ^15^, ^16^, ^17^ and rosiglitazone that induced RAI uptake and reduced serum thyroglobulin levels in some patients with DTC, but not resulting in a clinically significant response on long‐term follow‐up.^18^

Subsequently, selumetinib, a MEK1‐2 inhibitor, was studied as a potential treatment to reverse refractoriness to RAI, attaining a clinically meaningful increase in iodine uptake.^19^ Recently, Brown et al. (SEL‐I‐METRY trial)^20^ and Ho et al. (NCT02393690) have led two phase 2 trials with selumetinib followed by RAI, with results from both trials still pending publication. One final study of a similar nature, recently presented at the European Society for Medical Oncology Congress 2021, tested trametinib in patients with RAS‐mutated RR‐DTC. Authors observed cases of some restoration of RAI uptake and a 20% tumor response rate by the Response Evaluation Criteria in Solid Tumors (RECIST) v1.1 criteria (NCT03244956).^21^

Acctually there are 126 clinical trials on going in thyroid cancer that use as one of their arm radioidine with or without other treatments in combination (www.clinicaltrials.gov accessed on July, 7th 2022). Twenty seven of them evaluate a MKI, 5 trials study the combination of radioiodine with a MKI and 3 trials analyze the potential to resensitisize RR‐DTC cells to radioiodine therapy (al studies are summarize in Table 1). However, there are no published case reports using concomitant lenvatinib and RAI in RR‐DTC.

**TABLE 1:** Summary of clinical trials evaluating MKIs in DTC

NCT.Number	Phase	Title	Interventions	Outcome measures
NCT02211222	Expanded Access	An Expanded Access Program With Lenvatinib for the Treatment of Radioiodine‐Refractory Differentiated Thyroid Cancer	Lenvatinib	NA
NCT04619316	Phase 2	Enhancing Radioiodine Incorporation Into Radio Iodine Refractory Thyroid Cancers With MAPK Inhibition (ERRITI)	Trametinib 2 mg and Dabrafenib 75 mg (2–0‐2)	Proportion of patients with sufficiently increased tumoral iodine incorporation
NCT03690388	Phase 3	A Study of Cabozantinib Compared With Placebo in Subjects With Radioiodine‐refractory Differentiated Thyroid Cancer Who Have Progressed After Prior Vascular Endothelial Growth Factor Receptor (VEGFR) ‐Targeted Therapy	Cabozantinib vs Placebo	Progression Free Survival (PFS)
NCT03732495	Phase 2	Study of the Efficacy of Lenvatinib Combined With Denosumab in the Treatment of Patients With Predominant Bone Metastatic Radioiodine Refractory Differentiated Thyroid Carcinomas (LENVOS)	Lenvatinib + Denosumab	Determination of the efficacy of lenvatinib associated with denosumab in the treatment of patients with predominant bone metastases from Radioiodine‐Refractory Differentiated Thyroid Carcinoma
NCT03469011	Phase 1	A Study to Try to Bring Back Radioiodine Sensitivity in Patients With Advanced Thyroid Cancer.	Imatinib	Restore iodine uptake|Decrease overall tumor burden
NCT05182931	Phase 2	A Prospective, Multi‐Centre Trial of TKI Redifferentiation Therapy in Patients With RAIR Thyroid Cancer (I‐FIRST Study)	Dabrafenib 75 mg and Trametinib 2 mg	Progression free survival as assessed by RECIST 1.1 criteria at 6 months in participants who proceed to I131 treatment
NCT03533361	Expanded access	Expanded Access Program With Lenvatinib for the Treatment of Differentiated Thyroid Cancer in Brazil	Lenvatinib	NA
NCT02041260	Phase 2	A Phase II Trial of Cabozantinib for the Treatment of Radioiodine (RAI)‐Refractory Differentiated Thyroid Carcinoma (DTC) in the First‐line Setting	Cabozantinib	Number of Adverse Events
NCT02966093	Phase 3	A Trial of Lenvatinib (E7080) in Radioiodine (131 I)‐Refractory Differentiated Thyroid Cancer in China	Lenvatinib vs Placebo	Progression‐free survival (PFS)
NCT04544111	Phase 2	PDR001 Combination Therapy for Radioiodine‐Refractory Thyroid Cancer	Trametinib, Dabrafenib and PDR001	Overall response rate
NCT03573960	Phase 4	A Study to Evaluate the Safety and Efficacy of Lenvatinib in Participants With Refractory Differentiated Thyroid Cancer	Lenvatinib	Percentage of Participants with Grade 3 or Higher Treatment‐emergent Adverse Events (TEAEs)
NCT04061980	Phase 2	Encorafenib and Binimetinib With or Without Nivolumab in Treating Patients With Metastatic Radioiodine Refractory BRAF V600 Mutant Thyroid Cancer	Binimetinib, Encorafenib and Nivolumab	Overall response rate (ORR)
NCT00784303	Phase 2	Evaluating the Safety and Efficacy of Oral Lenvatinib in Medullary and Iodine‐131 Refractory, Unresectable Differentiated Thyroid Cancers, Stratified by Histology	Lenvatinib	Objective Response Rate (ORR)
NCT02152995	Phase 2	Trametinib in Increasing Tumoral Iodine Incorporation in Patients With Recurrent or Metastatic Thyroid Cancer	Iodine I‐124, Iodine I‐131 and Trametinib	Proportion of patients alive following treatment with trametinib and I‐124 (Cohort A) |Iodine incorporation in thyroid cancer metastases to a predicted lesional absorbed radiation dose equal to or exceeding 2000 cGy with the administration of ≤300 mCi radioiodine (RAI) (Cohort B) |Proportion of patients alive without disease progression (Cohort C)
NCT02973997	Phase 2	Lenvatinib and Pembrolizumab in Differentiated Thyroid Cancers (DTC)	Lenvatinib and Pembrolizumab	Complete response rate (Cohort 1)|Confirmed response rate (Cohort 2)
NCT01811212	Phase 2	Cabozantinib‐S‐Malate in Treating Patients With Refractory Thyroid Cancer	Cabozantinib S‐malate	Objective Response Rate, Defined as the Proportion of Patients Who Have Had a PR or CR as Assessed by the RECIST Version (v)1.1
NCT03914300	Phase 2	Testing the Combination of Cabozantinib, Nivolumab, and Ipilimumab (CaboNivoIpi) for Advanced Differentiated Thyroid Cancer	Cabozantinib S‐malate, Ipilimumab and Nivolumab	Objective response rate|Incidence of adverse events (AEs)|Duration of response|Progression‐free survival|Overall survival
NCT02702388	Phase 2	A Trial of Lenvatinib (E7080) in Subjects With Iodine‐131 Refractory Differentiated Thyroid Cancer to Evaluate Whether an Oral Starting Dose of 18 Milligram (mg) Daily Will Provide Comparable Efficacy to a 24 mg Starting Dose, But Have a Better Safety Profile	Lenvatinib	Objective Response Rate (ORR) as of Week 24 (ORR24wk)
NCT00510640	Phase 2	Thyroid Cancer and Sunitinib (THYSU)	Sunitinib	Objective response rate (ORR)
NCT03167385	Phase 2	Phase 2 Trial of Apatinib Mesylate in Locally Advanced/Metastatic Differentiated Thyroid Carcinoma	Apatinib Mesylate	Disease control rate
NCT04554680	Phase 2	Clinical Trial in RAI‐Refractory Thyroid Carcinoma Evaluating BRAF and MEK Blockade for Re‐differentiation Therapy	Dabrafenib and Trametinib	The proportion of participants attaining at least one tumor lesion with lesional dosimetry of ≥2000 cGy with I‐131 dose of ≤300 mCi
NCT04952493	Phase 2	Anlotinib or Penpulimab in Combination With RAI for DTC	Anlotinib hydrochloride, Sodium Iodide I 131 and Penpulimab	Objective response rate (ORR)
NCT02393690	Phase 2	Iodine I‐131 With or Without Selumetinib in Treating Patients With Recurrent or Metastatic Thyroid Cancer	Iodine I‐131, Placebo and Selumetinib	Response at 6 Months
NCT04858867	Phase 2	Reinducing Radioiodine‐sensitivity in Radioiodine‐refractory DTC Using Lenvatinib (RESET)	rhTSH‐stimulated I‐124, Intra‐therapeutic I‐131 and Lenvatinib	Fraction of RAI‐R thyroid cancer patients who are eligible for I‐131 therapy after 6‐ or 12‐week lenvatinib treatment
NCT03506048	Phase 2	Lenvatinib and Iodine Therapy in Treating Patients With Radioactive Iodine‐Sensitive Differentiated Thyroid Cancer	Iodine I‐131 and Lenvatinib	Efficacy of lenvatinib pretreatment along with radioactive iodine (RAI) in patients with previously treated RAI sensitive thyroid cancer
EudraCT 2015–002269‐47^a^	Phase 2	Investigating the potential clinical benefit of Selumetinib in resensitising advanced iodine refractory differentiated thyroid cancer to radioiodine therapy. SELIMETRY	Iodine I‐131 and Selumetinib	Potential for Selumetinib to resensitize RR‐DTC cells to radioiodine therapy
NCT03244956	Phase 2	Efficacy of MEK (Trametinib) and BRAFV600E (Dabrafenib) Inhibitors With Radioactive Iodine (RAI) for the Treatment of Refractory Metastatic Differentiated Thyroid Cancer	Iodine I‐131, Trametinib and Dabrafenib	Objective Response Rate (ORR) in metastatic radioactive Iodine Refractory Thyroid Cancer patients with RAS or BRAF mutation

^a^ This trial has no NCT number.

## 4 FUTURE TREATMENT SCENARIO

In spite of this lack of information, we can envision two potential scenarios where lenvatinib could be used concomitantly with RAI, both, at least in theory, opening treatment possibilities:

1. Patients with some, but not all, RAI‐avid lesions, particularly when ^18^FDG‐PET uptake is present.
2. Patients with RR‐DTC in whom some resensitization to RAI after treatment with lenvatinib could be observed.

In these situations, the combination of lenvatinib and RAI could improve disease outcomes in relevant highly selected patient groups. The first group of patients would have a mixed disease that may benefit from both types of treatment, while each treatment alone would be expectedly less effective, especially considering this disease would be considered RAI‐refractory according to current standards. The second group of patients with potential resensitization to RAI observed in some lesions but not in others, would be in a similar context to the previous group despite receiving the combined treatment with a different phasing. Nevertheless, the combination of lenvatinib and RAI needs to be validated in a proof‐of‐concept clinical trial before it can be confidently used.

## 5 DISCUSSION

As mentioned above, in some patients RAI is concentrated in some lesions, but not in others where ^18^FDG‐PET uptake is frequently seen. This scenario is propitious to expect benefit from combining an MKI with RAI, provided both types of RAI‐avid and ^18^FDG‐avid lesions coexist. Other patients who could benefit are those whose lesions are all RAI‐avid while still not cured after several RAI treatments. In these patients, the best response expected from RAI alone is a disease stabilization according to RECIST criteria.

In addition, the limited effect of the beta‐emission ray of RAI suggests that the combination with MKIs can also provide benefit in early disease stages and might not need to be restricted to progressive and iodine‐refractory disease.^22^

In turn, anti‐angiogenic MKIs normalize abnormal vascularization and decrease tumor growth, a dual mechanism that could improve the efficacy of RAI in eradicating disease, especially in RAI‐avid lesions potentially leading to prolonged PFS and overall survival.^22^

Furthermore, by adding a cytostatic agent to a cytotoxic treatment, one could improve disease control even after stopping the MKI, provided the sustained cytotoxic effect of RAI is maintained.^22^

Sorafenib has, however, been studied in this context but results were negative in its capacity to show any reinduction of RAI uptake in RR‐DTC patients.^23^  Sorafenib and lenvatinib have different kinome‐inhibition profiles, and this difference supports the idea of lenvatinib still deserving being tested in these population.

## 6 CONCLUSION

Currently limited treatment options for RR‐DTC patients make further research on effective drugs essential. If, in the meantime, these new strategies become available to the clinic, some patients could benefit from potential combinations of already effective therapies such as RAI and MKIs.

In conclusion, the combination of lenvatinib with RAI has demonstrated efficacy and low toxicity in preclinical studies that warrant further clinical research in patients with RR‐DTC, or even in the earlier stages of the disease. Results from ongoing clinical trials in these patient populations are eagerly awaited.

## FUNDING INFORMATION

The author received an honorarium payment from Eisai Farmacéutica SA in line with ICMJE guidelines.

## CONFLICT OF INTEREST

Dr Urbano Anido Herranz has received research funding, honoraria, and non‐financial or other support from Advanced Accelerator Applications, Astellas, Bayer, BMS, EUSA, Ipsen, Janssen, Kyowa Kirin, Lilly, Novartis, Pfizer, Pierre Fabre, Roche, Sanofi.

## Data Availability

Data sharing is not applicable to this article as no new data were created or analyzed in this study.
